# 

**Published:** 1907-09-15

**Authors:** 


CONTENTS

Event and Comment - - - - - - - - - 431
Treatment of Pyorrhea, - *By Grace Pearl Rogers, D.D.S.* 434
Porcelain Jacket Crowns, - - - *By John E. Roche, D.D.S.* 454
The Evolution of the "Inlay" in Dentistry,
*By F. W. Macdonald, D.D.S.* 456
A Case of Actinomycosis, - *By George Zederbaum, D.D.S.* 460
The Pulp and Its Pathology, *By Russell W. Bunting, D.D.S.* 468
Sanitation of Cement for Fillings, Crowns and Bridges,
*By M. J. Emelin, D.D.S.* 472
Electro-Gilding, - - - *By J. Tenney Barker, D.D.S.* 474
Bibliography - - - - - - - - - - 478
Indents - - - - - - - - - - - - 480

